# Fluorophore Labeled Kinase Detects Ligands That Bind within the MAPK Insert of p38α Kinase

**DOI:** 10.1371/journal.pone.0039713

**Published:** 2012-07-02

**Authors:** Matthäus Getlik, Jeffrey R. Simard, Martin Termathe, Christian Grütter, Matthias Rabiller, Willem A. L. van Otterlo, Daniel Rauh

**Affiliations:** 1 Chemical Genomics Centre of the Max Planck Society, Dortmund, Germany; 2 Fakultät Chemie, Chemische Biologie, Technische Universität Dortmund, Dortmund, Germany; 3 Department of Chemistry and Polymer Sciences, Stellenbosch University, Stellenbosch, South Africa; 4 Max-Planck-Institute of Molecular Physiology, Dortmund, Germany; Albert-Ludwigs-University, Germany

## Abstract

The vast majority of small molecules known to modulate kinase activity, target the highly conserved ATP-pocket. Consequently, such ligands are often less specific and in case of inhibitors, this leads to the inhibition of multiple kinases. Thus, selective modulation of kinase function remains a major hurdle. One of the next great challenges in kinase research is the identification of ligands which bind to less conserved sites and target the non-catalytic functions of protein kinases. However, approaches that allow for the unambiguous identification of molecules that bind to these less conserved sites are few in number. We have previously reported the use of fluorescent labels in kinases (FLiK) to develop direct kinase binding assays that exclusively detect ligands which stabilize inactive (DFG-out) kinase conformations. Here, we present the successful application of the FLiK approach to develop a high-throughput binding assay capable of directly monitoring ligand binding to a remote site within the MAPK insert of p38α mitogen-activated protein kinase (MAPK). Guided by the crystal structure of an initially identified hit molecule in complex with p38α, we developed a tight binding ligand which may serve as an ideal starting point for further investigations of the biological function of the MAPK insert in regulating the p38α signaling pathway.

## Introduction

During the last decade, a plethora of small molecule kinase modulators have been developed [1]. The majority of these molecules bind to the highly conserved ATP pocket and are often plagued by modest selectivity, which in the case of kinase inhibitors leads to the inhibition of multiple kinases [2], [3]. Thus, selective modulation of kinase function remains a major task and the identification of next generation ligands which bind to less conserved sites and specifically target non-catalytic functions of kinases such as protein-protein interactions, DNA binding and subcellular localization, are considered the next great challenges in kinase research [19]. Allosteric ligands that bind to distal binding sites and often stabilize catalytically inactive kinase conformations are prime examples of modern kinase inhibitor research and represent a promising approach towards the development of selective kinase inhibitors [4]. However, only a few kinases such as PDK1, Akt, Mek or Bcr-Abl are known to be regulated by such ligands [1], [3], [5], [6], [7] and approaches that allow for the unambiguous identification of molecules that bind to these less conserved sites are few in number.

**Figure 1:**
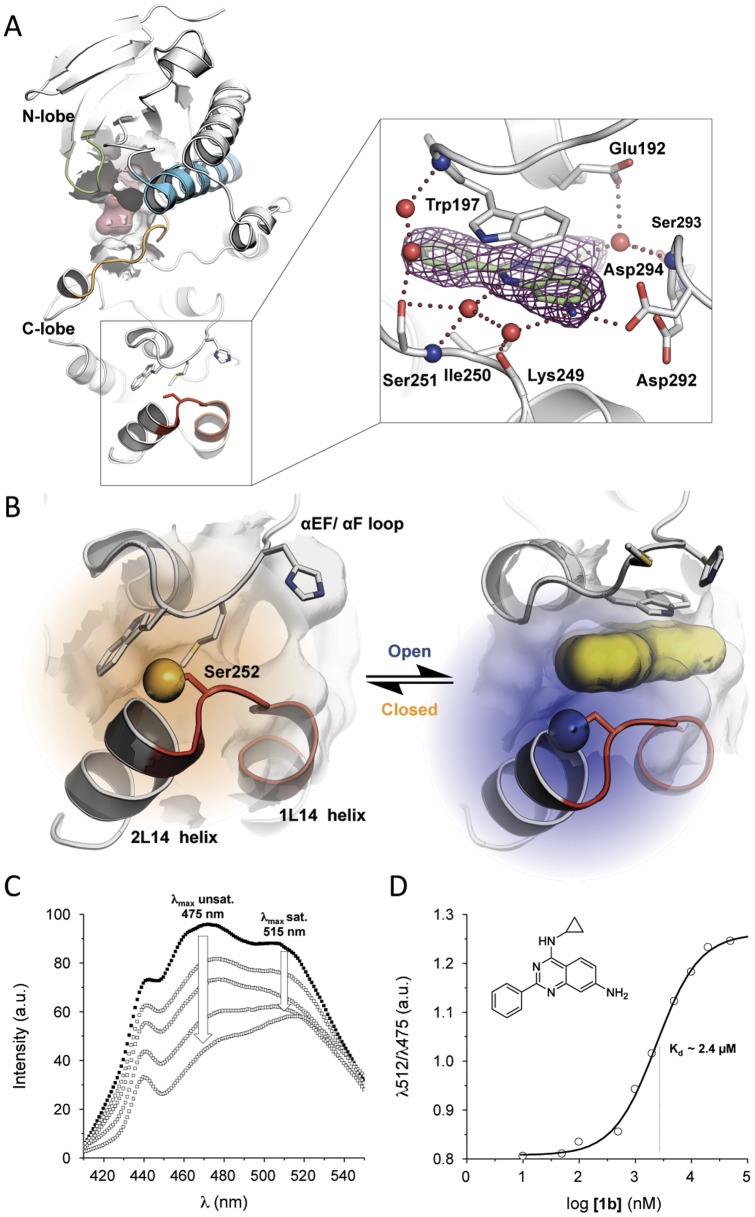
Crystal structure of p38α and lipid binding FLiK-assay. (A) Crystal structures of wild type p38α (grey) in complex with ATP (red) and in complex with **1b** (green, boxed). The alpha-helix C (blue), activation loop (orange), glycine-rich loop (green) and, MAPK insert (red) are highlighted. **1b** binds to the lipid binding site and forms a direct hydrogen bond with Asp294. Furthermore, binding of the ligand is stabilized through a network of water-mediated hydro-gen bonds as well as π-π-interactions between the quinazoline core and the side chain of Trp197. (B) Conformational changes triggered by ligand binding to the p38α MAPK insert. Ser252 was mutated to Cys252 and was labeled with a fluorophore (large spheres) to generate a direct binding assay that detects structural changes in the conformation of the lipid binding site. (C) Acrylodan emission at 475 nm decreases upon binding of **1b** to the lipid binding site of p38α. (D) Endpoint equilibrium measurements can be made to directly obtain the K_D_ of **1b**.

Interestingly, several MAP kinases, cyclin dependant kinases (CDKs) and glycogen synthase kinase 3 (GSK-3) contain a hydrophobic pocket at their C terminus about 30 Å away from the ATP-pocket [8], [9]. This C-terminal insert regulates the intracellular localization of GSK-3, binds regulatory proteins in CDK2 and has been shown to bind substrates such as transcription factors and phosphatases in Erk2 [9]. Recently, Diskin *et al*. confirmed the existence of the MAPK insert in p38α using protein X-ray crystallography and proposed that this structural feature may “fine-tune” kinase activity.[10] This same binding site in p38α is formed between the α-helices 1L14 and 2L14, and the C-terminal domain of the kinase (Figure 1A). Structural biology studies of p38α in complex with β-octyl-glucopyranoside revealed flexibility of this region and its ability to accommodate small molecules. Binding of the detergent shifts the two α-helices 1L14 and 2L14 about 3 Å away from the C-lobe and induces a conformational change of the αEF/αF-loop (Figure 1B) [10]. Although, as yet no clear biological function can be attributed to this hydrophobic pocket in p38α, potent ligands which specifically bind to this allosteric site may offer a valuable starting point for the development of chemical biology tool compounds for the investigation of its biological function.

## Results

The lipid binding pocket of p38α has been reported to bind lipids [10] as well as a few small molecule fragments. Interestingly, these reported ligands do not bind exclusively to the MAPK insert, as structural studies have revealed secondary binding within the ATP-pocket [9], [11], [12]. To our knowledge, no high affinity compounds are known which exclusively bind to the lipid binding site of p38α. Thus, identification of new ligands with high affinity for this pocket might stimulate the development of more specific ligands which may enable future research to elucidate its function. To this end, we applied the FLiK approach to develop a fluorescent-labeled kinase assay system, which specifically takes advantage of the ligand-induced conformational change of α-helices 1L14 und 2L14 of the p38α MAPK insert. This HTS-amenable assay allowed the identification and characterization of 2-phenylquinazolines as ligands for this allosteric site. We were able to further develop higher affinity ligands and confirmed binding to this remote pocket using protein X-ray crystallography.

We initially screened a small molecule library using our previously reported FLiK assay, which detects ligand-induced conformational changes in the glycine-rich loop of p38α, [13] and weakly detected binding of the 2-phenylquinazoline **1b** to p38α. To better understand the binding mode and how this ligand interacts with the glycine-rich loop, we crystallized **1b** in complex with wild type p38α and were surprised to find the molecule bound exclusively within the p38α MAPK insert (Figure 1A) (PDB code 4DLI), far away from the glycine-rich loop. The bound ligand is stabilized by a complex network of interactions. The primary amine in the 7-position of the quinazoline core directly hydrogen bonds to the side chain of Asp294. The quinazoline core itself forms interactions with the peptide backbone of Lys249 and Ile250 via two mediating water molecules. Furthermore, the side chain of Trp197 stabilizes binding of the quinazoline core via π-π-interactions. Interestingly, the side chain of Ser251 forms two water-mediated hydrogen bonds to the peptide backbone of Trp197 that may further stabilize the conformation of the αEF/αF-loop and the flipped orientation of the Trp197 side chain which is adopted when the ligand is bound. The cyclopropyl substituent occupies a hydrophobic subpocket that is formed by the side chains of Leu195, Leu232, Leu236 and Leu291 in the back of this binding pocket. Finally, the secondary amine which bridges the cyclopropyl residue to the quinazoline core interacts with the side chain of Glu192 and the peptide backbone of both Leu291 and Asp292 via water-mediated hydrogen bonds. However, in order to accurately measure the affinity of these compounds and to understand structure-activity relationships, it was necessary to develop a direct binding assay to enable specific detection of ligands binding within the MAPK insert. We sought to take advantage of the flexibility of this site in response to ligand binding and applied the FLiK approach to this pocket by introducing a Cys mutation (S252C) via site-directed mutagenesis into the flexible loop bridging α-helices 1L14 and 2L14, to enable subsequent labeling of the Cys side chain with the environmentally-sensitive fluorophore acrylodan (Figure 1B). We expected that conformational changes induced by ligand binding to this site would change the conformation of the MAP insert and, consequently, the charged microenvironment in the vicinity of acrylodan. Indeed, the altered fluorescent properties resulted in a shift of the emission maxima in the ligand bound state (Figure 1C). Upon ligand binding to this site, the ratio of fluorescence intensities measured at these maxima changed in a dose-dependent manner, allowing direct determination of the binding constant K_D_ (Figure 1D). Using this assay, the initial hit **1b** bound with a K_D_ of 2.4 μM to the p38α MAPK insert. SB203580 and BIRB-796, known p38α inhibitors which bind within the ATP binding site, were not detected up to a concentration of 10 µM (*data not shown*) confirming that the observed fluorescence response is due solely to binding to the p38α MAPK insert.

**Figure 2:**
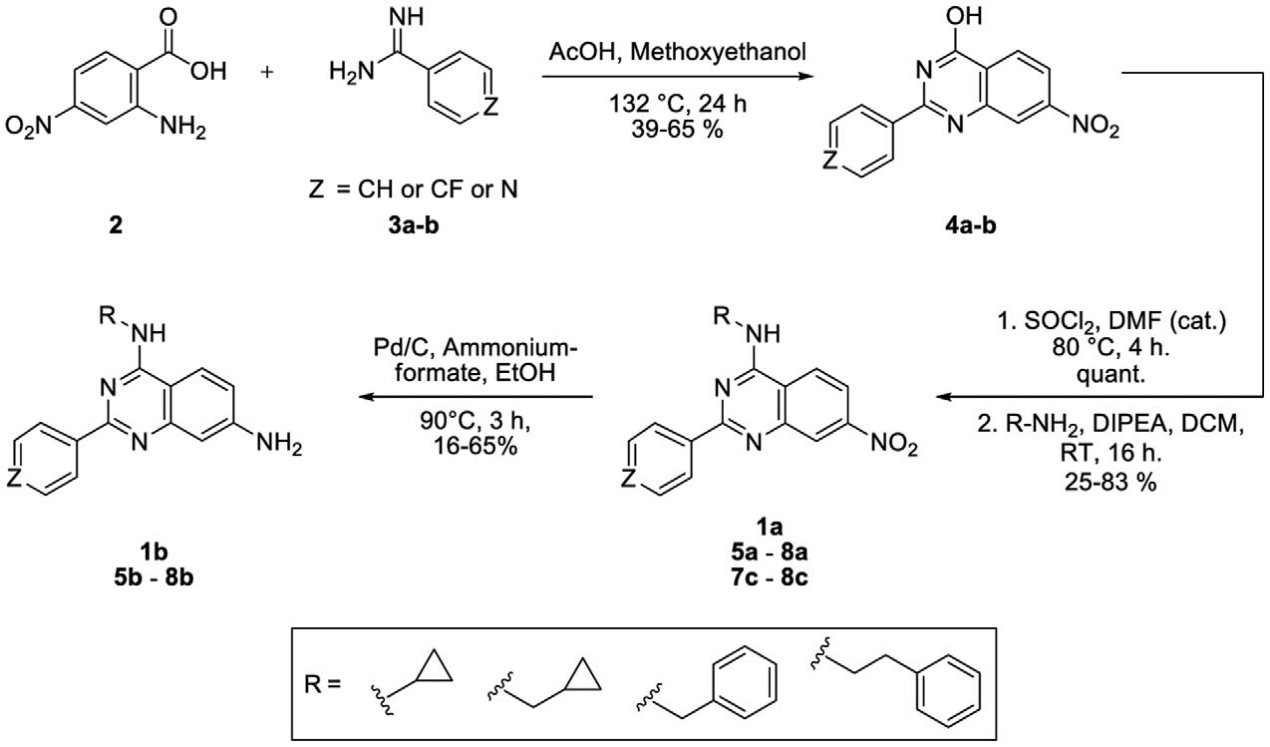
General synthesis of 2-arylquinazolines. See table in Figure 3 for identity of substituents (R).

**Figure 3:**
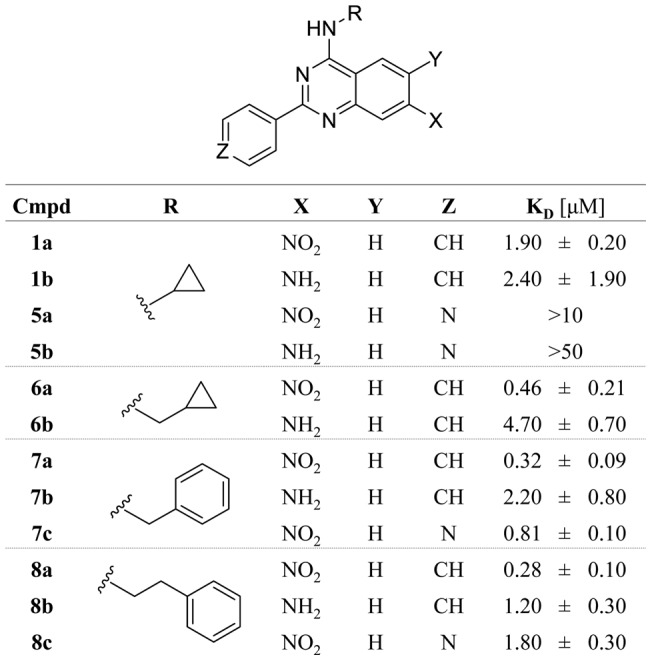
Structure activity relationship of 2-arylquinazolines. Each concentration of compound was tested in triplicate. Ratiometric emission values were plotted against the concentration of compound on a logarithmic scale to generate a binding curve. K_D_ values of various 2-arylquinazolines were determined bound to p38α.

**Figure 4:**
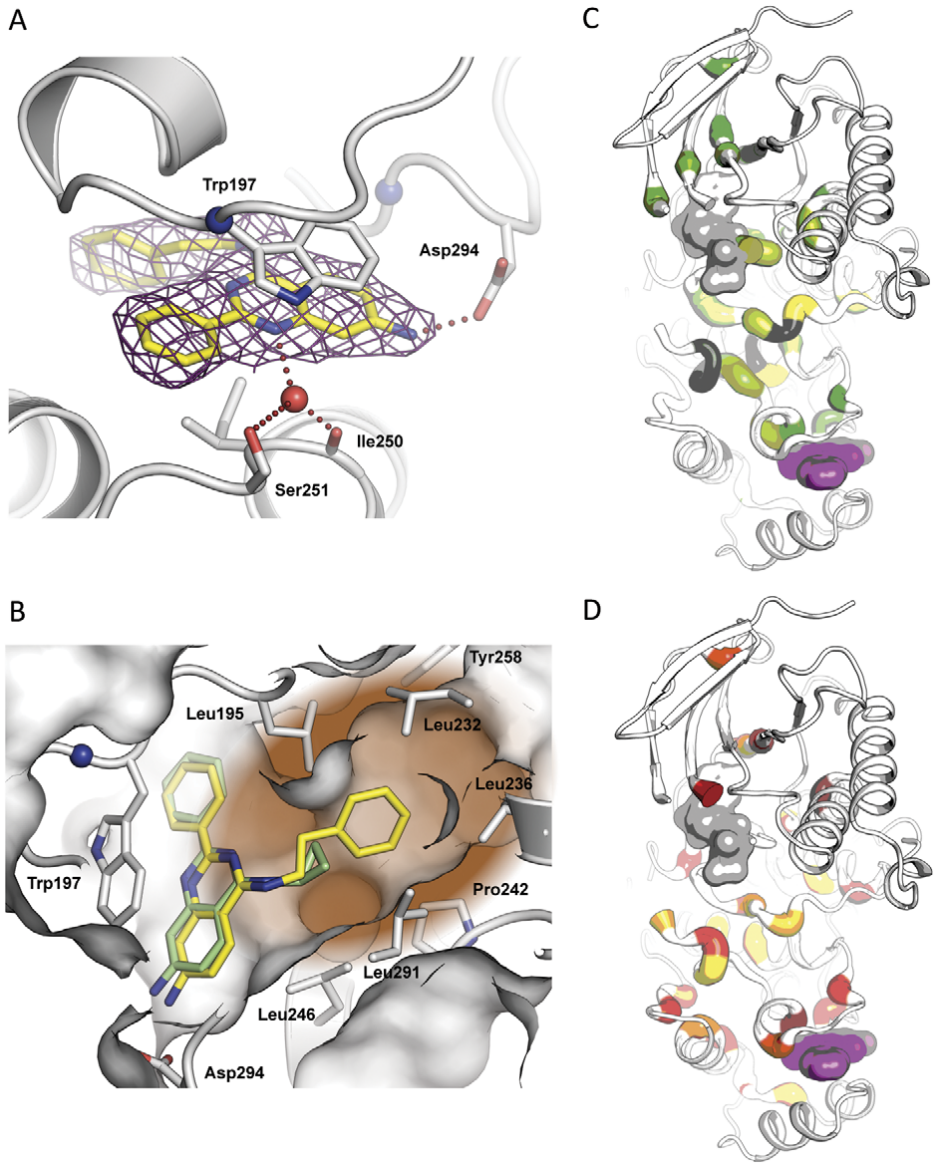
Co-crystal structure of p38α and co-evolution analysis. (A) Crystal structure of wild type p38α (grey) in complex with **8b** (yellow). (B) Alignment of **1b** and **8b** bound to the lipid binding site of MAPK p38α. The 2-phenyl quinazoline scaffolds align nicely. The phenyl ethylene moiety of **8b** extends further into the hydro-phobic back pocket (brown) resulting in a higher binding affinity. (C, D) p38α in complex with **1b**. Amino acids showing significant co-evolution are colored according to the strength of the cumulative effect they exert on other residues (C) and of the cumulative effect other residues have on them (D), yellow symbolizing the strongest effect on both panels.

Guided by the crystal structure of **1b** in complex with p38α we synthesized a focused set of 2-arylquinazolines designed to probe the MAP insert pocket and increase compound binding affinity within this site (Figure 2). We hypothesized that this cavity would tolerate larger and more hydrophobic side chains at the 4-position of the quinazoline core. Briefly, the quinazoline cores were generated by a Niementowski-type strategy which entailed condensing amidines with the appropriate amino carboxylic acids. Position-4 of the resultant quinazolinols was functionalized through chlorination using SOCl_2_, thereby enabling the subsequent introduction of primary amines to this position via nucleophilic substitution. Finally, the nitro groups on the quinazoline scaffolds were reduced utilizing Pd/C and ammonium formate as a hydrogen donor, to afford the corresponding 4,6-quinazolinediamines. The resulting SAR of this focused set of ligands followed the predicted trend with **8a** being the tightest binder with a K_D_ of 280 nM (Figure 3). As revealed by protein X-ray crystallography of the corresponding amine analog **8b** (PDB code 4DLJ), the phenylethylene moiety extends further into the hydrophobic pocket when compared to **1b**, suggesting that the deeper occupancy of this hydrophobic sub-pocket by the large substituent in the 4-position likely contributes to the increased affinity of **8a** (Figure 4B). This also explains the slight loss of potency of **7a** and **6a**, which have progressively shorter moieties at this position. Interestingly, all corresponding amine analogs bound to the lipid binding pocket with lower affinities than their corresponding nitro analogs. Also, introduction of a pyridine in the 2-position of the quinazoline core decreased the binding affinity. Due to the hydrophobic nature of the MAPK insert in p38α, we speculate that the introduction of polar groups or heteroatoms might be less tolerated. Although several co-crystallization attempts were unsuccessful, we postulate that the nitro-substituent may increase affinity by forming favorable water- or ion-mediated interactions with Asp residues 292 and 294. Considering the unique binding mode of **1b**, we wanted to better understand why this compound was initially detected, although weakly, and initially identified by our glycine-rich loop FLiK binding assay. We carried out a co-evolution analysis of the sequences of several mammalian CMGC kinases [14] using the analysis algorithms previously described by Lockless and Ranganathan [15]. This analysis supports the existence of a network of interdependent amino acids which link the hydrophobic pocket to the N-lobe of CMGC kinases, thus providing evidence of a clear correlation between residues in the ATP pocket of p38α and its MAPK insert (Figure 4C, D). Interestingly, this analysis is supported by NMR data recently reported by Francis *et al*. in which the binding of the p38α DFG-out inhibitor BIRB-796 not only perturbed chemical shifts within the ATP binding site but also affected the chemical shifts of residues in the MAPK insert in the C-terminal lobe [16]. While it is important to note that this analysis merely detects evolutionary correlations, the internal “hydrophobic spine” highlighted by this analysis, and proposed elsewhere, [17], [18] appears to be well in line with our experimental results.

## Discussion

**Figure 5:**
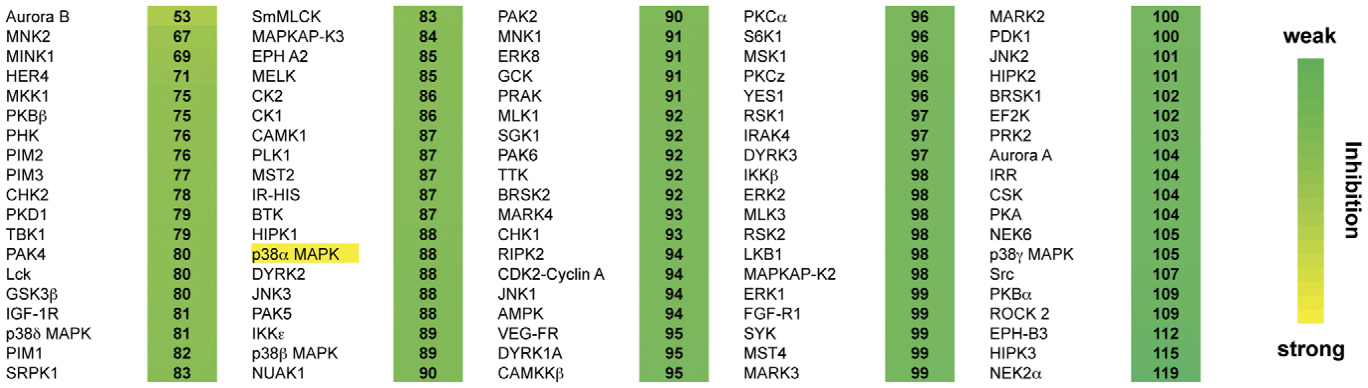
Kinase selectivity profile of 1b. Compound **1b** was profiled against a selected panel of 95 different kinases at a concentration of 10 µM employing a radioactive (^33^P-ATP) filter-binding assay (National Centre for Protein Kinase Profiling, Dundee). The numbers represent the remaining kinase activity in %. The profiling reveals that compound **1b** does not inhibit enzymatic activity of any of the tested kinases.

Although initial detection of **1b** with a binding assay based on conformational changes of the glycine-rich loop supports this “cross-talk” model of allosteric modulation, we were not able to observe any inhibition of kinase activity when testing this focused library of MAP insert binders on p38α. Additionally, **1b** was profiled against 95 additional kinases in activity-based biochemical assays (Figure 5). Interestingly, there are naturally occurring isoforms of p38α which show altered signaling pathway preferences [19]. The major differences between these isoforms and wild type p38α lie at the C-terminal end of the kinase, including regions within or proximal to the MAP kinase insert. We propose that the MAP kinase insert may have a scaffolding function and somehow regulates p38α signaling independent of the activity of p38α itself [20]. Although it is beyond the scope of this work, we believe that future studies aimed at elucidating the biological function(s) of this site may rely on the use of tightly binding molecules, such as those presented, which can perturb the conformation of the MAP kinase insert, disrupt or enhance protein-protein interactions with this structural feature and provide kinase researchers with alternative mechanisms for modulating the complex MAPK network [21], [22]. In conclusion, we have developed a fluorescence-based HTS binding assay for the MAPK insert of p38α and identified scaffolds which bind exclusively to this unique pocket. Using protein X-ray crystallography, we validated the screening results and successfully guided the medicinal chemistry efforts to generate potent binders of the p38α lipid binding site. These compounds might present a valuable starting point for further investigations of the p38α MAPK insert biology. Also, the presented assay is a further proof of the general applicability of the FLiK technology and presents an advantageous strategy for the identification of yet unknown kinase modulators.

## Materials and Methods

The fluorophore 6-acryloyl-2-dimethylaminonaphthalene (acrylodan) was purchased from Invitrogen GmbH (Germany). Cuvettes and mini stir bars were obtained from Carl Roth GmbH (Germany). Small volume (20 µL fill volume) black flat bottom 384-well plates were obtained from Greiner Bio-One GmbH (Solingen, Germany). Crystallization plates (EasyXtal Tool; 24-well) were obtained from Qiagen GmbH (Germany). All supplies for the p38α HTRF assay kit were purchased from CisBio (Bagnols-sur-Cèze, France).

### Expression, purification and labeling of p38α

The human p38α kinase construct containing the mutations required for specific labeling (C119S/C162S/S252C) as well as wild type p38α were cloned into a pOPINF vector and transformed as an N-terminal His-tag construct with a PreScission Protease cleavage site into BL21(DE3) Codon+RIL *E. coli*. Protein expression, purification, fluorophore labeling and verification of mono-labeling of the kinase were performed essentially as described previously. Labeling of the mutant p38α protein with thiol-reactive acrylodan was carried out by adding a 1.5∶1 molar excess of the fluorophore (dissolved in DMSO) to the purified protein in 50 mM Hepes Buffer (pH 7.0). The mixture was allowed to react in the dark overnight at 4°C. Unreacted acrylodan was subsequently removed by three cycles of buffer exchange into FLiK Measurement Buffer (50 mM Hepes Buffer, pH 7.4+200 mM NaCl) using a 10k-MWCO Centricon. The protein was concentrated to ∼200 μM, aliquoted and frozen at −80°C. Mono-labeling of the protein was verified by ESI-MS as described previously [23], [24].

**Table 1:** Data Collection and Refinement Statistics for p38α crystal structures in complex with **1b** and **8b** a.

	p38αwith 1b (4DLI)	p38αwith 8b (4DLJ)	
**Data collection**			
Space group	P2_1_2_1_2_1_	P2_1_2_1_2_1_	
Cell dimensions			
*a*, *b*, *c* (Å)	66.26, 74.22, 77.92	65.98, 74.51, 78.32	
α, β, γ (°)	90.00, 90.00, 90.00	90.00, 90.00, 90.00	
Resolution (Å)	45.0–1.91 (2.00–1.91)^a^	45.0–2.60 (2.70–2.60)^a^	
*R*_sym_ or *R*_merge_ (%)	4.0 (40.1)	7.6 (35.9)	
*I*/σ*I*	22.0 (4.0)	17.0 (4.3)	
Completeness (%)	99.5 (99.8)	99.8 (99.7)	
Redundancy	3.97 (4.03)	4.34 (3.36)	
		
**Refinement**			
Resolution (Å)	41.7–1.91	39.2–2.60	
No. reflections	30473	12383	
*R*_work_/*R*_free_	21.5/25.5	21.0/31.4	
No. atoms			
Protein	2685	2697	
Ligand/ion	42	55	
Water	135	52	
*B*-factors	32.1	30.8	
Protein	32.0	30.8	
Ligand/ion	28.7	35.4	
Water	35.5	25.7	
R.m.s. deviations			
Bond lengths (Å)	0.015	0.016	
Bond angles (°)	1.455	1.551	
**Data collection details**			
Wavelength (Å)	1.00000	1.54170	
Temperature	90K	100K	
X-ray source	Synchrotron radiation	Bruker AXS Microstar	
**Ramachandran Plot**			
Residues in most favored regions	87.7%	90.2%	
Residues in additional allowed regions	11.7%	8.8%	
Residues in generously allowed regions	0.6%	1.0%	
Residues in disallowed regions	0.0%	0.0%	

a For all p38α complex structures, diffraction data from one crystal was used to determine the structure. Values in parenthesis are for the highest resolution shell.

### Characterization of the p38α lipid lite FLiK assay

Characterization of the fluorescence response of MAP insert-labeled p38α was carried out as described previously for p38α labeled on the activation loop [23]. For this particular FLiK assay, we employed **1b** as a reference compound since protein X-ray crystal structures revealed exclusive binding to the lipid binding site of p38α. The binding of this ligand induces movement in the MAP insert, thereby altering the microenvironment and the fluorescence properties of acrylodan. Briefly, a polystyrene cuvette containing a suspension of 50 nM acrylodan-labeled p38α in FLiK Buffer was excited at 386 nm and emission spectra were measured in the absence and presence of increasing concentrations of **1b**. The compound was dissolved in 100% DMSO and delivered in sequential small doses to a single sample. The %v/v DMSO did not exceed 0.2% in cuvettes. These measurements revealed that the major emission maximum shifts from ∼475 nm to ∼515 nm as the lipid acrylodan-labeled lipid binding sites become saturated with **1b**. This shift in emission maxima enabled the use of ratiometric values (R = I_515_/I_475_) for plotting binding curves and determining the K_D_ of ligands. Ratiometric fluorescence readouts are advantageous in that they provide an internal correction for small errors in sample volume which can affect signal intensity. All measurements of the cuvettes were made with a JASCO FP-6500 fluorescence spectrophotometer (JASCO GmbH, Germany).

### Adaptation to high-throughput screening formats

The assay was adapted to high-throughput screening formats which enabled K_D_ values to be determined in black small volume assay plates. Briefly, a dilution series of each compound was first prepared in 100% DMSO at 20X the desired final concentration and 1 μL was transferred to the assay plate. Compounds were then mixed with 19 µL of FLiK Buffer (supplemented with 0.01% Triton X-100) containing acrylodan-labeled p38α (100 nM). The final concentration of compound in the dilution series ranged from 0–50 μM (5% v/v DMSO final). Plates were covered with an adhesive aluminum foil and incubated for 30 min at RT prior to measurement of emission intensities at 470 and 520 nm using a Tecan Safire^2^ plate reader. The adjustment in chosen wavelengths in 384-well plates was necessary to maximize the resulting fluorescence ratio as we suspect the addition of detergent to assay buffer caused a slight shift in the emission maxima. Binding curves were plotted using the ratio R = I_520_/I_470_ to determine the K_D_. A calculated Z'>0.7 (using negative and positive binding controls) suggests that the FLiK assay for the lipid site of p38α is robust and amenable to high-throughput screening formats.

### Chemistry

Unless otherwise noted, all reagents and solvents were purchased from Acros, Fluka, Sigma, Aldrich or Merck and used without further purification. Dry solvents were purchased as anhydrous reagents from commercial suppliers. ^1^H and ^13^C NMR spectra were recorded on a Varian Mercury 400, Bruker Avance DRX 400, Bruker DRX 500 or Varian Inova 600 spectrometer. ^1^H NMR spectroscopy chemical shifts are reported in δ (ppm) as s (singlet), d (doublet), dd (doublet of doublet), t (triplet), q (quartet), m (multiplet) and bs (broad singlet), and are referenced to the residual solvent signal: CDCl_3_ (7.26), CD_3_OD (4.87) and, DMSO-*d6* (2.50). ^13^C NMR spectra are referenced to the residual solvent signal: CDCl_3_ (77.0), CD_3_OD (21.4), DMSO-*d6* (39.0). All final compounds were purified to >95% purity, as determined by high-performance liquid chromatography (HPLC). Purities were measured using an Agilent 1200 Series HPLC system with UV detection at 210 nm (System: Agilent Eclipse XDB-C18 4.6×150 mm, 5 µM, 10 to 100% CH_3_CN in H_2_O, with 0.1% TFA, for 15 min at 1.0 mL/min). High resolution electrospray ionization mass spectra (ESI-FTMS) were recorded on a Thermo LTQ Orbitrap (high resolution mass spectrometer from Thermo Electron) coupled to an ‘Accela’ HPLC System supplied with a ‘Hypersil GOLD’ column (Thermo Electron). Analytical TLC was carried out on Merck 60 F245 aluminum-backed silica gel plates. Compounds were purified by column chromatography using Baker silica gel (40–70 µm particle size). Preparative HPLC was conducted on a Varian HPLC system (Pro Star 215) with a VP 250/21 Nucleosil C18 PPN column from Macherey-Nagel and monitored by UV at λ = 254 nm. Microwave-assisted reactions were carried out with a ‘Discover’ system from CEM Corporation.

#### Synthesis of 7-Nitro-2-phenyl-4(3*H*)-quinazolinone 4a

Benzamidine **3a** (6.0 g, 50 mmol) and 4-nitro-2-aminobenzoic acid **2** (2.8 g, 16 mmol) were dissolved in 2-methoxyethanol (300 mL) after which acetic acid (3.6 mL, 63 mmol) was added. The solution then heated at reflux (132°C) for 20 h with stirring. The reaction mixture was then cooled, filtered and the filtrate washed several times with cold MeOH to afford the desired product **4a** (2.8 g, 65%) as a green-yellow colored solid which was utilized in other reactions without further purification. **^1^H-NMR** (400 MHz, DMSO-*d6*): *δ* 12.90 (s, 1H), 8.42 (d, *J* = 2.2 Hz, 1H), 8.36 (d, *J* = 8.7 Hz, 1H), 8.24–8.19 (m, 3H), 7.66–7.55 (m, 3H); **^13^C-NMR** (101 MHz, DMSO-*d6*): *δ* 162.29, 155.45, 152.20, 149.93, 132.92, 132.89, 129.59, 129.06, 128.91, 126.14, 123.16, 120.96; **HRMS** (ESI-MS): Calculated: 268.07167 for C_14_H_10_N_3_O_3_ [M+H]^+^, Found: 268.07173.

#### Synthesis of N-Cyclopropyl-7-nitro-2-phenyl-4-quinazolinamine 1a

7-Nitro-2-phenyl-4(3*H*)-quinazolinone **4a** (100 mg, 0.373 mmol) was transferred to a dry flask under argon after which thionyl chloride (2 mL) and 1–2 drops DMF were added. The reaction was then heated at reflux (80°C) for 3 h after which the solvent was removed under vacuum. This afforded the respective chloride as a brown-colored solid which was used without any further purification in the following reaction. The chloride (100 mg, 0.35 mmol) was dissolved in dry DCM (4 mL) and cyclopropylamine (37 μL, 0.52 mmol) in *i*-PrOH (2 mL) was then added drop-wise. The reaction mixture was then stirred at rt for 24 h, after which the solvent was removed under vacuum. The resulting residue was subsequently purified by silica gel column chromatography (3% MeOH/DCM as elution solvent) to afford the desired product **1a** as a yellow solid (89 mg, 83%). **^1^H-NMR** (400 MHz, DMSO-*d_6_*) δ 8.69 (d, *J* = 3.6 Hz, 1H), 8.54 (dd, *J* = 6.6, 3.0 Hz, 2H), 8.44 (dd, *J* = 10.7, 5.7 Hz, 2H), 8.16 (dd, *J* = 9.0, 2.3 Hz, 1H), 7.56–7.48 (m, 3H), 3.27–3.21 (m, 1H), 0.95–0.86 (m, 2H), 0.77–0.68 (m, 2H); **^13^C-NMR** (101 MHz, DMSO-*d_6_*) δ 161.22, 160.58, 149.94, 149.90, 137.82, 130.72, 128.33, 128.19, 125.23, 122.80, 118.34, 117.33, 24.48, 6.13. **HRMS** (ESI-MS): Calculated: 307.11895 for C_17_H_14_O_2_N_4_ [M+H]^+^, Found: 307.11895.

#### Synthesis of N-(Cyclopropylmethyl)-7-nitro-2-phenyl-4-quinazolinamine 6a

The reaction was performed as described above for **1b**, in that the required chloride (100 mg, 0.373 mmol) in dry DCM (2 mL) was reacted with cyclopropylmethylamine (44.95 μL, 0.53 mmol) *i*-PrOH (2 mL) to afford the desired product **6a** as a yellow solid (47.0 mg, 42%) after purification by silica gel column chromatography (DCM as eluent). **^1^H-NMR** (400 MHz, DMSO-*d_6_*): δ 8.88 (br s, 1H), 8.52 (d, *J* = 9.0 Hz, 1H), 8.50–8.45 (m, 2H), 8.43 (d, *J* = 2.4 Hz, 1H), 8.19 (dd, *J* = 9.0, 2.4 Hz, 1H), 7.54–7.51 (m, 3H), 3,57–3.54 (m, 2H), 1.35–1.22 (m, 1H), 0.57–0.48 (m, 2H), 0.40–0.34 (m, 2H); **^13^C-NMR** (101 MHz, DMSO-*d_6_*): δ 161.15, 159.36, 150.05, 149.87, 137.71, 130.80, 128.42, 128.13, 125.32, 122.64, 118.45, 117.43, 45.50, 10.50, 3.71; **HRMS** (ESI-MS): Calculated: 321.13460 for C_18_H_17_N_4_O_2_ [M+H]^+^, Found: 321.13449.

#### Synthesis of N-Benzyl-7-nitro-2-phenyl-4-quinazolinamine 7a

The reaction was performed as described above with **1b**, in that the required chloride (100 mg, 0.373 mmol) in dry DCM (2 mL) was reacted with benzylamine (57.29 μL, 0.525 mmol) in *i*-PrOH (2 mL) to afford the desired product **7a** as a yellow solid (104.6 mg, 84%) after silica gel column chromatography (DCM as elution solvent). **^1^H-NMR** (400 MHz, DMSO-*d_6_*): δ 9.39 (br s, 1H), 8.57 (d, *J* = 9.1 Hz, 1H), 8.49–8.44 (m, 3H), 8.24 (dd, *J* = 9.1, 2.3 Hz, 1H), 7.53–7.45 (m, 5H), 7.36–7.32 (m, 2H), 7.26–722 (m, 1H), 4.94 (d, *J* = 5.7 Hz, 2H); **^13^C-NMR** (101 MHz, DMSO-*d_6_*): δ 161.15, 159.38, 150.19, 137.61, 134.64, 130.87, 129.53, 129.20, 128.45, 128.19, 127.56, 127.03, 125.30, 122.77, 118.73, 117.47, 44.17; **HRMS** (ESI-MS): Calculated: 357.13460 for C_21_H_17_N_4_O_2_ [M+H]^+^, Found: 357.13438.

#### Synthesis of 7-Nitro-2-phenyl-N-(2-phenylethyl)-4-quinazolinamine 8a

The reaction was performed as described above with **1b**, in that the required chloride (100 mg, 0.373 mmol) in dry DCM (2 mL) was reacted with phenethylamine (66.13 μL, 0.525 mmol) in *i*-PrOH (2 mL) to afford the desired product **8a** as a yellow solid (111.5 mg, 86%) after silica gel column chromatography (DCM as elution solvent). **^1^H-NMR** (400 MHz, DMSO-*d_6_*): δ 8.94 (br s, 1H), 8.52–8.42 (m, 4H), 8.18 (dd, *J* = 9.0, 2.3 Hz, 1H), 7.54–7.52 (m, 3H), 7.34–7.28 (m, 4H), 7.24–7.18 (m, 1H), 3.91–3.85 (m, 2H), 3.06 (t, *J* = 7.5, 2H); **^13^C-NMR** (101 MHz, DMSO-*d_6_*): δ 161.03, 159.28, 150.03, 149.53, 139.41, 137.52, 130.89, 128.76, 128.47, 128.42, 128.15, 126.24, 125.18, 122.48, 118.60, 117.38, 42.68, 34.29; **HRMS** (ESI-MS): Calculated: 371.15025 for C_22_H_19_N_4_O_2_ [M+H]^+^, Found: 371.15014.

#### Synthesis of N^4^-Cyclopropyl-2-phenyl-4,7-quinazolinediamine 1b

***N***-Cyclopropyl-7-nitro-2-phenyl-4-quinazolinamine **1a** (32 mg, 0.10 mmol) and ammonium formate (40 mg, 0.60 mmol) were dissolved in absolute EtOH (3 mL). A spatula tip of Pd/C (5%) was then added and the reaction mixture was heated at reflux (90°C) for 2 h. The reaction solution was cooled and filtered through celite, and the filtrate evaporated under vacuum. Purification of the resulting residue was performed by preparative HPLC (Eluent: 0.1% TFA in MECN/H_2_O) to afford **1b** as a yellow solid (18 mg, 65%). **^1^H-NMR** (400MHz, DMSO-*d_6_*) δ 8.48 (dd, *J* = 7.5, 2.0 Hz, 2H), 7.89 (d, *J* = 8.6 Hz, 1H), 7.81 (s, 1H), 7.56–7.42 (m, 3H), 6.83–6.68 (m, 2H), 5.87 (s, 2H), 3.19–3.12 (m, 1H), 0.89–0.76 (m, 2H), 0.70–0.60 (m, 2H); **^13^C-NMR** (101 MHz, CD_3_OD) δ 161.29, 157.35, 156.29, 141.56, 133.29, 131.95, 129.15, 128.63, 125.17, 117.52, 101.94, 97.39, 25.17, 5.97. **HRMS** (ESI-MS): Calculated: 277.14477 for C_17_H_17_N_4_ [M+H]^+^, Found: 277.14484.

#### Synthesis of N^4^-(Cyclopropylmethyl)-2-phenyl-4,7-quinazolinediamine 6b

The reduction was performed as described above for **1b**, utilizing *N*–(cyclopropylmethyl)–7–nitro-2–phenyl–4–quinazolinamine **6a** (46 mg, 0.14 mmol) and ammonium formate (54.5 mg, 0.864 mmol) to afford compound **6b** as a yellow solid (16 mg, 43%) after silica gel column chromatography (1.5% MeOH in DCM as eluent). **^1^H-NMR** (400 MHz, DMSO-*d_6_*): δ 8.42 (dd, *J* = 8.0, 1.5 Hz, 2H), 7.91 (d, *J* = 8.8 Hz, 1H), 7.87–7.84 (m, 1H), 7.50–7.39 (m, 3H), 6.76 (dd, *J* = 8.8, 2.2 Hz, 1H), 6.73 (d, *J* = 2.1 Hz, 1H), 5.80 (s, 2H), 3.49 (t, *J* = 6.2 Hz, 2H), 1.28–1.21 (m, 1H), 0.51–0.44 (m, 2H), 0.36–0.30 (m, 2H); **^13^C-NMR** (101 MHz, DMSO-*d_6_*): δ 159.21, 159.07, 152.59, 151.83, 139.25, 129.60, 128.07, 127.70, 123.66, 115.44, 106.26, 104.65, 62.08, 11.11, 3.59; **HRMS** (ESI-MS): Calculated: 291.16042 for C_18_H_19_N_4_ [M+H]^+^, Found: 291.16007.

#### Synthesis of N^4^-Benzyl-2-phenyl-4,7-quinazolinediamine 7b

The reduction was performed as described above for **1b**, utilizing *N*–benzyl–7–nitro-2–phenyl–4–quinazolinamine **7a** (40 mg, 0.11 mmol) and ammonium formate (42.4 mg, 0.67 mmol) in EtOH (8 mL) to afford compound **7b** as a yellow solid (16 mg, 44%) after silica gel column chromatography (1% MeOH/DCM as eluent). **^1^H-NMR** (400 MHz, DMSO-*d_6_*): δ 8.40–8.34 (m, 3H), 7.95 (d, *J* = 8.8 Hz, 1H), 7.46–7.39 (m, 5H), 7.32–7.29 (m, 2H), 7.23–7.20 (m, 1H), 6.78 (dd, *J* = 8.8, 2.2 Hz, 1H), 6.74 (d, *J* = 2.1 Hz, 1H), 5.84 (s, 2H), 4.85 (d, *J* = 5.8 Hz, 2H); **^13^C-NMR** (101 MHz, DMSO-*d_6_*): δ 159.03, 158.97, 152.63, 152.06, 140.48, 139.17, 129.49, 128.17, 127.96, 127.64, 127.32, 126.56, 123.53, 115.55, 106.29, 104.63, 43.50; (ESI-MS): Calculated: 327.16042 for C_21_H_19_N_4_ [M+H]^+^, Found: 327.16018.

#### Synthesis of 2-Phenyl-N^4^-(2-phenylethyl)-4,7-quinazolinediamine 8b

The reduction was performed as described for **1b** above, with 7–nitro-2–phenyl–*N*–(2–phenylethyl)–4–quinazolinamine **8a** (48 mg, 0.12 mmol) and ammonium formate (48.8 mg, 0.77 mmol) in EtOH (8 mL) to afford compound **8b** as a yellow solid (15 mg, 34%) after silica gel column chromatography (1.5% MeOH in DCM as eluent). **^1^H-NMR** (400 MHz, DMSO-*d_6_*): δ 8.46 (dd, *J* = 7.9, 1.5 Hz, 2H), 7.97–7.92 (m, 1H), 7.87 (d, *J* = 8.8 Hz, 1H), 7.52–7.42 (m, 3H), 7.34–7.31 (m, 4H), 7.25–7.18 (m, 1H), 6.79–6.72 (m, 2H), 5.86 (s, 2H), 3.84–3.78 (m, 2H), 3.02 (t, *J* = 7.5 Hz, 2H); **^13^C-NMR** (101 MHz, DMSO-*d_6_*): δ 159.10, 159.03, 157.64, 151.82, 139.89, 139.25, 129.64, 128.74, 128.44, 128.10, 127.69, 126.10, 123.57, 115.51, 106.22, 104.68, 42.29, 35.05; **HRMS** (ESI-MS): Calculated: 341.17607 for C_22_H_21_N_4_ [M+H]^+^, Found: 341.17574.

#### Synthesis of 7-Nitro-2-(4-pyridinyl)-4(3H)-quinazolinone 4b

Pyridine-4-carboximidamide **14** (1.50 g, 9.5 mmol), 4-nitro-2-aminobenzoic acid (0.57 g, 3.1 mmol) and acetic acid (0.97 mL, 17 mmol) were dissolved in 2-methoxyethanol (20 mL) and the solution heated at reflux (132°C) for 20 h with stirring, as described above for the synthesis of **4a**. Work-up as before afforded the desired product **4b** (0.479 g, 57%) as a brown-colored solid, which was used without further purification. **^1^H-NMR** (400 MHz, DMSO-*d_6_*): δ 13.18 (s, 1H), 8.83 (d, *J* = 6.0 Hz, 2H), 8.49 (d, *J* = 2.1 Hz, 1H), 8.40 (d, *J* = 8.7 Hz, 1H), 8.29 (dd, *J* = 8.7, 2.1 Hz, 1H), 8.14 (d, *J* = 6.1 Hz, 2H); **^13^C-NMR** (101 MHz, DMSO-*d_6_*): δ 161.24, 152.79, 151.39, 150.14, 149.66, 139.70, 128.30, 125.89, 122.66, 121.91, 120.93; **HRMS** (ESI-MS): Calculated: 269.06692 for C_13_H_9_N_4_O_3_ [M+H]^+^, Found: 269.06696.

#### Synthesis of N-Cyclopropyl-7-nitro-2-(4-pyridinyl)-4-quinazolinamine 5a

The reaction was performed as described above for the synthesis of compound **1a**, with 7-nitro-2-(4-pyridinyl)-4(3*H*)-quinazolinone **4b** (300 mg, 1.05 mmol) being used to generated the respective chloride, after which cyclopropylamine (219 μL, 3.15 mmol) was used with the addition of an aliquot of NEt_3_ (218 μL, 1.57 mmol), to afford the desired product **5a** as a yellow oil (80 mg, 25%) after column chromatography (1% MeOH/DCM as eluent). **^1^H-NMR** (400 MHz, DMSO-*d_6_*): δ 8.82 (br d, *J* = 3.6 Hz, 1H), 8.76 (d, *J* = 5.7 Hz, 2H), 8.50–8.47 (m, 2H), 8.37–8.35 (m, 2H), 8.23 (dd, *J* = 9.0, 2.3 Hz, 1H), 3.29–3.22 (m, 1H), 0.96–0.90 (m, 2H), 0.78–0.73 (m, 2H); **^13^C-NMR** (101 MHz, DMSO-d6): δ 160.83, 159.47, 150.17, 150.05, 149.56, 145.04, 125.32, 122.99, 121.90, 119.23, 117.73, 24.53, 6.07; **HRMS** (ESI-MS): Calculated: 308.11420 for C_16_H_14_N_5_O_2_ [M+H]^+^, Found: 308.11422.

#### Synthesis of N-Benzyl-7-nitro-2-(4-pyridinyl)-4-quinazolinamine 7c

The reaction was performed as described above for the synthesis of compound **1a**, with 7-nitro-2-(4-pyridinyl)-4(3*H*)-quinazolinone **4b** (100 mg, 0.373 mmol) used to generated the respective chloride after which benzylamine (114.6 μL, 1.05 mmol) to afford the desired product **7c** as a yellow solid (66 mg, 51%) after column chromatography (1% MeOH/DCM as eluent, followed by a second purification utilizing 60% EtOAc/petroleum ether as solvent). **^1^H-NMR** (400 MHz, DMSO-*d_6_*): δ 9.47 (br t, *J* = 5.7 Hz, 1H), 8.74 (d, *J* = 5.7 Hz, 2H), 8.60 (d, *J* = 9.0 Hz, 1H), 8.52 (d, *J* = 2.2 Hz, 1H), 8.32–8.27 (m, 3H), 7.48 (d, *J* = 7.4 Hz, 2H), 7.36–7.33 (m, 2H), 7.27–7.24 (m, 1H), 4.95 (d, *J* = 5.5 Hz, 2H); **^13^C-NMR** (101 MHz, DMSO-*d_6_*): δ 159.57, 159.42, 150.21, 149.81, 144.91, 138.86, 128.39, 127.53, 126.99, 125.33, 123.06, 121.84, 119.50, 117.85, 117.80, 44.21; **HRMS** (ESI-MS): Calculated: 358.12985 for C_20_H_16_N_5_O_2_ [M+H]^+^, Found: 358.12955.

#### Synthesis of 7-Nitro-N-(2-phenylethyl)-2-(4-pyridinyl)-4-quinazolinamine 8c

The reaction was performed as described above for the synthesis of compound **1a**, with 7-nitro-2-(4-pyridinyl)-4(3*H*)-quinazolinone **4b** (100 mg, 0.373 mmol) used to generated the respective chloride after which phenylethylamine (132 μL, 1.05 mmol) was utilized to afford the desired product **8c** as a yellow solid (70 mg, 54%) after column chromatography (1% MeOH in DCM, followed by a second column using 60% EtOAc: petroleum ether as solvent). **^1^H-NMR** (400 MHz, DMSO-*d_6_*): δ 9.01–8.98 (m, 1H), 8.77 (d, *J* = 5.6 Hz, 2H), 8.51–8.48 (m, 2H), 8.33 (d, *J* = 5.4 Hz, 2H), 8.27 (dd, *J* = 9.0, 1.9 Hz, 1H), 7.34–7.28 (m, 4H), 7.22–7.18 (m, 1H), 3.93–3.90 (m, 2H), 3.07 (t, *J* = 7.4 Hz, 2H); **^13^C-NMR** (101 MHz, DMSO-*d_6_*): δ 159.52, 159.40, 150.13, 149.69, 145.09, 139.30, 128.72, 128.38, 126.17, 125.20, 123.01, 121.85, 119.37, 117.82, 117.76, 42.56, 34.25; **HRMS** (ESI-MS): Calculated: 372.14550 for C_21_H_18_N_5_O_2_ [M+H]^+^, Found: 372.14528.

#### Synthesis of N^4^-Cyclopropyl-2-(4-pyridinyl)-4,7-quinazolinediamine 5b

The reduction was performed as described above for the synthesis of **1b**, with *N*-cyclopropyl-7-nitro-2-(4-pyridinyl)-4-quinazolinamine **5a** (55 mg, 0.179 mmol) and ammonium formate (67.7 mg, 1.07 mmol) in EtOH (5 mL) and the reaction mixture being heated at reflux (90°C) for 3 h. After a similar work-up by filtration, purification was carried out by preparative HPLC (0.1% TFA in H2O/MeCN). The combined fractions were concentrated under vacuum and then made basic with NEt3. This was followed by an extraction of the aqueous phase with EtOAc (4 times) and subsequent drying of the organic phase with Na_2_SO_4_. After filtration and evaporation of the solvent under vacuum the desired product **5b** was isolated as a yellow solid (8.0 mg, 16%). 1H-NMR (400 MHz, MeOH-d4): δ 8.65 (d, J = 4.6 Hz, 2H), 8.39 (d, J = 4.6 Hz, 2H), 7.81 (d, *J* = 8.8 Hz, 1H), 6.96–6.85 (m, 2H), 1.15 (br d, *J* = 6.1 Hz, 1H), 0.92–0.89 (m, 2H), 0.73–0.68 (m, 2H); **^13^C-NMR** (101 MHz, MeOH-*d_4_*): δ 162.58, 159.92, 154.35, 152.96, 150.21, 149.33, 124.51, 124.09, 118.15, 107.77, 107.03, 25.05, 7.16; **HRMS** (ESI-MS): Calculated: 278.14002 for C_16_H_16_N_5_ [M+H]^+^, Found: 278.14004.

### Protein crystallography

Various inhibitors were co-crystallized with wild type p38α using conditions similar to those described previously [23]. Briefly, protein-inhibitor complexes were prepared by mixing 40 µL p38α (10 mg/mL) with 0.4 µL of inhibitor (100 mM in DMSO) and incubating the mixture for 1 h on ice. Samples were centrifuged at 13,000 rpm for 5 min to remove excess inhibitor. In some cases, co-crystallization carried out in the presence of excess inhibitor improved occupancy of the compounds in the crystal structure. Crystals were grown in 24-well crystallization plates using the hanging drop vapor diffusion method and by mixing 1.5 µL protein-inhibitor solution with 0.5 µL reservoir (100 mM MES pH 5.6–6.2, 20–30% PEG4000 and 50 mM n-octyl-β-D-glucopyranoside). Crystallization plates (EasyXtal Tool; 24-well) were obtained from Qiagen GmbH (Hilden, Germany). Cuvettes and mini stir bars were obtained from Carl Roth GmbH (Karlsruhe, Germany).

### Structure determination, refinement and crystallographic statistics

For the crystals of p38α with inhibitors 20% glycerol was used as cryo protectant before they were flash frozen in liquid nitrogen. Diffraction data of the p38α-**1b** complex crystals was measured at the PX10SA beam line of the Swiss Light Source (PSI, Villingen, Switzerland) using a wavelength close to 1 Å. Diffraction data of the p38α-**8b** complex was collected in-house. Both datasets were processed with XDS and scaled using XSCALE [25]. Thep38α-inhibitor complex structures were solved by molecular replacement with PHASER [26] using the published p38α structures (PDB code: 1ZYJ) [27] or (PDB code: 2EWA) [28] as templates. The molecules in the asymmetric unit were manually modified using the program COOT [29]. The model was first refined with CNS8 using simulated annealing to reduce model bias. The final refinement was performed with REFMAC5 [30]. Inhibitor topology files where generated using the Dundee PRODRG2 server [31]. The two refined structures were validated with PROCHECK [32]. Data collection, structure refinement statistics, PDB-ID codes and further details for the data collection as well as Ramachandran plot results are shown in Table 1. PyMOL was used to produce the figures [33].
